# Cyclo­oxygenase-1-selective inhibitor SC-560

**DOI:** 10.1107/S1600536809001779

**Published:** 2009-01-23

**Authors:** Sihui Long, Kathryn L. Theiss, Tonglei Li, Charles D. Loftin

**Affiliations:** a Department of Pharmaceutical Sciences, College of Pharmacy, University of Kentucky, Lexington, KY 40536, USA

## Abstract

In the title compound, 5-(4-chloro­phen­yl)-1-(4-methoxy­phen­yl)-3-(trifluoro­meth­yl)-1*H*-pyrazole (SC-560), C_17_H_12_ClF_3_N_2_O, a COX-1-selective inhibitor, the dihedral angles between the heterocycle and the chlorobenzene and methoxybenzene rings are 41.66 (6) and 43.08 (7)°, respectively. The dihedral angle between the two phenyl rings is 59.94 (6)°. No classic hydrogen bonds are possible in the crystal, and intermolecular interactions must be mainly of the dispersion type. This information may aid the identification of dosage formulations with improved oral bioavailability.

## Related literature

For background literature, see: Choi *et al.* (2008 ▶); Cusimano *et al.* (2007 ▶); Kundu & Fulton (2002 ▶); Penning *et al.* (1997 ▶); Smith *et al.* (2000 ▶); Teng *et al.* (2003 ▶); Tiano *et al.* (2002 ▶); For related structures, see: Allen (2002 ▶); Charlier *et al.* (2004 ▶); Norris *et al.* (2005 ▶); Sonar *et al.* (2004 ▶); Zhu *et al.* (2004 ▶).
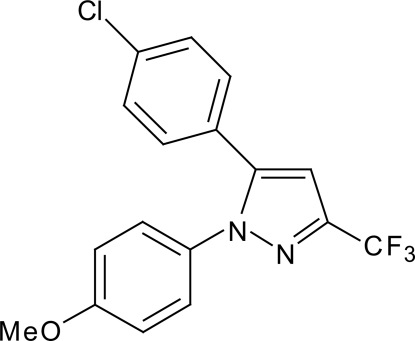


## Experimental

### 

#### Crystal data

C_17_H_12_ClF_3_N_2_O
*M* *_r_* = 352.74
Monoclinic, 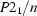

*a* = 15.585 (3) Å
*b* = 7.1671 (14) Å
*c* = 15.789 (3) Å
β = 116.81 (3)°
*V* = 1574.1 (5) Å^3^
*Z* = 4
Mo *K*α radiation
μ = 0.28 mm^−1^
*T* = 90 (2) K
0.20 × 0.10 × 0.10 mm

#### Data collection

Nonius KappaCCD diffractometer
Absorption correction: multi-scan (*SCALEPACK*; Otwinowski & Minor, 1997 ▶) *T*_min_ = 0.946, *T*_max_ = 0.972
6895 measured reflections
3608 independent reflections
3030 reflections with *I* > 2σ(*I*)
*R*_int_ = 0.020

#### Refinement

*R*[*F* ^2^ > 2σ(*F* ^2^)] = 0.035
*wR*(*F* ^2^) = 0.094
*S* = 1.04
3608 reflections
218 parameters
H-atom parameters constrained
Δρ_max_ = 0.34 e Å^−3^
Δρ_min_ = −0.29 e Å^−3^

### 

Data collection: *COLLECT* (Hooft, 1998 ▶); cell refinement: *DENZO-SMN* (Otwinowski & Minor, 1997 ▶); data reduction: *DENZO-SMN*; program(s) used to solve structure: *SHELXS97* (Sheldrick, 2008 ▶); program(s) used to refine structure: *SHELXL97* (Sheldrick, 2008 ▶); molecular graphics: *SHELXTL* (Sheldrick, 2008 ▶); software used to prepare material for publication: *SHELXTL* and local procedures.

## Supplementary Material

Crystal structure: contains datablocks global, I. DOI: 10.1107/S1600536809001779/pv2130sup1.cif

Structure factors: contains datablocks I. DOI: 10.1107/S1600536809001779/pv2130Isup2.hkl

Additional supplementary materials:  crystallographic information; 3D view; checkCIF report

## Figures and Tables

**Fig. 1.**
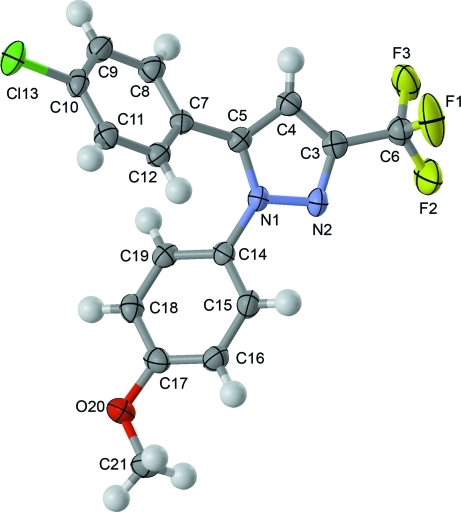
The molecular structure of (I), showing atom displacement ellipsoids at the 50% probability level.

**Fig. 2.**
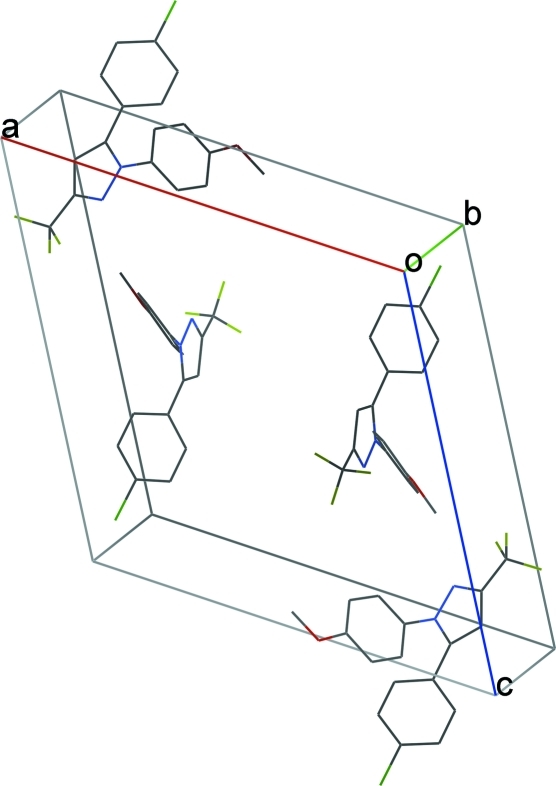
Crystal packing of SC-560.

## supplementary crystallographic information

### Comment

Inhibition of the two cyclooxygenase (COX) isoforms is considered the primary mechanism responsible for both the therapeutic and toxic effects of nonsteroidal anti-inflammatory drugs (NSAIDS) (Smith, *et al.*, 2000). Both of the COX isoforms have been shown to contribute to inflammation and tumor genesis, and therapeutic benefits may result from either COX-1 or COX-2 selective inhibition (Tiano, *et al.* 2002; Choi, *et al.* 2008; Kundu & Fulton, 2002; Cusimano, *et al.* 2007). The development of the COX-2-selective inhibitor celecoxib led to identification of a variety of structurally related compounds with varying selectivity for the COX isoforms, SC-560 was one of them (Penning, *et al.* 1997; Choi, *et al.* 2008). However, SC-560's poor bioavailability may limit its effects (Teng, *et al.* 2003). The information from crystal structure may provide direction in suitable dosage formulation. Herein, we describe the first crystal structure of SC-560, (I), a selective and potent inhibitor of the cyclooxygenase-1 isoform (Penning, *et al.* 1997).

The crystal structure of (I) is presented in Fig. 1. The structure lacks the moieties necessary for hydrogen-bonding to occur between molecules (Fig. 2). Thus, the lattice energy mainly consists of dispersion energies, which typically result in a low melting point because of the weak intermolecular interactions (as confirmed by melting point measurement). Despite the entire chemical structure being fused together by three aromatic rings, a large conjugate system between the rings is not seen due to steric repulsion between the two phenyl rings. Similar structures are abundant in the Cambridge Structural Database (CSD - Version 5.29; Allen, 2002), EYISAG (Sonar, *et al.* 2004), IZAYUD (Charlier, *et al.* 2004), JAQBIN (Norris, *et al.* 2005), and MAJGUA (Zhu, *et al.* 2004) are few of them. To conclude, the single-crystal structure of SC-560 was solved. Because there is no hydrogen bonding in the structure, the major contribution for the lattice energy stems from weak dispersion energies leading to its low melting point at 335.5 K.

### Experimental

Commercial SC-560 was dissolved in HPLC grade methanol in a glass vial at room temperature. The vial was sealed with Parafilm with numerous pin-size holes introduced to allow for evaporation of the solvent. Colorless block crystals were obtained following approximately one week of slow evaporation.

### Refinement

H atoms were found in difference Fourier maps and those on the aromatic ring subsequently placed in idealized positions with C—H distances of 0.95 Å and isotropic displacement parameters equal to 1.2*U*_eq_ of the attached carbon atom. Hydrogen atom coordinates in the methyl group were placed in idealized positions with C—H distances of 0.98 Å and isotropic displacement parameters of 1.5*U*_eq_ of the carbon atom.

### Crystal data

**Table:**

C_17_H_12_ClF_3_N_2_O	*F*(000) = 720
*M**_r_* = 352.74	*D*_x_ = 1.488 Mg m^−^^3^
Monoclinic, *P*2_1_/*n*	Mo *K*α radiation, λ = 0.71073 Å
*a* = 15.585 (3) Å	Cell parameters from 3880 reflections
*b* = 7.1671 (14) Å	θ = 1.0–27.5°
*c* = 15.789 (3) Å	µ = 0.28 mm^−^^1^
β = 116.81 (3)°	*T* = 90 K
*V* = 1574.1 (5) Å^3^	Block, colorless
*Z* = 4	0.20 × 0.10 × 0.10 mm

### Data collection

**Table:**

Nonius KappaCCD diffractometer	3608 independent reflections
Radiation source: fine-focus sealed tube	3030 reflections with *I* > 2σ(*I*)
graphite	*R*_int_ = 0.020
Detector resolution: 18 pixels mm^-1^	θ_max_ = 27.5°, θ_min_ = 1.5°
ω scans at fixed χ = 55°	*h* = −20→20
Absorption correction: multi-scan (*SCALEPACK*; Otwinowski & Minor, 1997)	*k* = −9→9
*T*_min_ = 0.946, *T*_max_ = 0.972	*l* = −20→20
6895 measured reflections	

### Refinement

**Table:**

Refinement on *F*^2^	Primary atom site location: structure-invariant direct methods
Least-squares matrix: full	Secondary atom site location: difference Fourier map
*R*[*F*^2^ > 2σ(*F*^2^)] = 0.035	Hydrogen site location: inferred from neighbouring sites
*wR*(*F*^2^) = 0.094	H-atom parameters constrained
*S* = 1.04	*w* = 1/[σ^2^(*F*_o_^2^) + (0.0421*P*)^2^ + 0.7994*P*] where *P* = (*F*_o_^2^ + 2*F*_c_^2^)/3
3608 reflections	(Δ/σ)_max_ = 0.004
218 parameters	Δρ_max_ = 0.34 e Å^−^^3^
0 restraints	Δρ_min_ = −0.29 e Å^−^^3^

### Special details

**Table:**

Geometry. All e.s.d.'s (except the e.s.d. in the dihedral angle between two l.s. planes) are estimated using the full covariance matrix. The cell e.s.d.'s are taken into account individually in the estimation of e.s.d.'s in distances, angles and torsion angles; correlations between e.s.d.'s in cell parameters are only used when they are defined by crystal symmetry. An approximate (isotropic) treatment of cell e.s.d.'s is used for estimating e.s.d.'s involving l.s. planes.
Refinement. Refinement of *F*^2^ against ALL reflections. The weighted *R*-factor *wR* and goodness of fit *S* are based on *F*^2^, conventional *R*-factors *R* are based on *F*, with *F* set to zero for negative *F*^2^. The threshold expression of *F*^2^ > σ(*F*^2^) is used only for calculating *R*-factors(gt) *etc*. and is not relevant to the choice of reflections for refinement. *R*-factors based on *F*^2^ are statistically about twice as large as those based on *F*, and *R*- factors based on ALL data will be even larger.

### Fractional atomic coordinates and isotropic or equivalent isotropic displacement parameters (Å^2^)

**Table:**

	*x*	*y*	*z*	*U*_iso_*/*U*_eq_	
Cl13	0.06492 (3)	0.90044 (6)	0.10611 (2)	0.02831 (12)	
N2	0.38203 (8)	0.87316 (17)	0.67980 (8)	0.0188 (2)	
O20	0.12419 (8)	0.14611 (15)	0.54981 (7)	0.0287 (3)	
F3	0.56584 (7)	1.19886 (15)	0.74065 (7)	0.0382 (3)	
F2	0.52162 (8)	1.04490 (15)	0.83041 (6)	0.0443 (3)	
N1	0.32413 (8)	0.81035 (16)	0.59070 (7)	0.0169 (2)	
C10	0.14398 (10)	0.89725 (19)	0.22720 (9)	0.0199 (3)	
C5	0.33171 (9)	0.91657 (19)	0.52240 (9)	0.0171 (3)	
C18	0.21726 (10)	0.3440 (2)	0.50702 (9)	0.0196 (3)	
H18	0.2135	0.2529	0.4618	0.023*	
F1	0.44389 (7)	1.28688 (15)	0.75784 (7)	0.0448 (3)	
C11	0.10734 (10)	0.8619 (2)	0.29082 (10)	0.0199 (3)	
H11	0.0408	0.8367	0.2688	0.024*	
C19	0.26912 (9)	0.50583 (19)	0.51716 (9)	0.0179 (3)	
H19	0.3033	0.5239	0.4809	0.021*	
C7	0.26748 (10)	0.89925 (18)	0.41982 (9)	0.0170 (3)	
C14	0.27105 (9)	0.64202 (19)	0.58060 (9)	0.0173 (3)	
C16	0.17333 (11)	0.4491 (2)	0.62713 (10)	0.0244 (3)	
H16	0.1419	0.4285	0.6657	0.029*	
C6	0.48912 (10)	1.1368 (2)	0.74825 (10)	0.0222 (3)	
C12	0.16930 (10)	0.86393 (19)	0.38715 (9)	0.0190 (3)	
H12	0.1449	0.8411	0.4315	0.023*	
C4	0.39888 (9)	1.0532 (2)	0.56958 (9)	0.0189 (3)	
H4	0.4216	1.1482	0.5428	0.023*	
C8	0.30233 (10)	0.9337 (2)	0.35415 (10)	0.0211 (3)	
H8	0.3689	0.9576	0.3757	0.025*	
C3	0.42585 (9)	1.0204 (2)	0.66545 (9)	0.0190 (3)	
C15	0.22267 (10)	0.6153 (2)	0.63461 (10)	0.0222 (3)	
H15	0.2231	0.7102	0.6768	0.027*	
C17	0.17048 (10)	0.3143 (2)	0.56306 (10)	0.0217 (3)	
C9	0.24066 (11)	0.9335 (2)	0.25715 (10)	0.0238 (3)	
H9	0.2645	0.9578	0.2124	0.029*	
C21	0.07544 (14)	0.1110 (3)	0.60633 (11)	0.0403 (5)	
H21A	0.1214	0.1185	0.6737	0.060*	
H21B	0.0468	−0.0138	0.5921	0.060*	
H21C	0.0248	0.2043	0.5919	0.060*	

### Atomic displacement parameters (Å^2^)

**Table:**

	*U*^11^	*U*^22^	*U*^33^	*U*^12^	*U*^13^	*U*^23^
Cl13	0.0288 (2)	0.0370 (2)	0.01330 (17)	0.00223 (16)	0.00434 (14)	0.00022 (14)
N2	0.0178 (6)	0.0216 (6)	0.0138 (5)	−0.0009 (5)	0.0044 (4)	−0.0028 (4)
O20	0.0349 (6)	0.0259 (6)	0.0258 (5)	−0.0125 (5)	0.0143 (5)	−0.0024 (4)
F3	0.0326 (5)	0.0472 (6)	0.0402 (5)	−0.0209 (5)	0.0211 (4)	−0.0206 (5)
F2	0.0533 (6)	0.0441 (6)	0.0177 (4)	−0.0183 (5)	0.0001 (4)	−0.0012 (4)
N1	0.0180 (5)	0.0186 (6)	0.0126 (5)	−0.0015 (5)	0.0057 (4)	−0.0014 (4)
C10	0.0238 (7)	0.0184 (7)	0.0134 (6)	0.0030 (5)	0.0046 (5)	0.0000 (5)
C5	0.0177 (6)	0.0180 (7)	0.0159 (6)	0.0022 (5)	0.0080 (5)	0.0011 (5)
C18	0.0207 (7)	0.0180 (7)	0.0178 (6)	0.0029 (5)	0.0067 (5)	−0.0008 (5)
F1	0.0342 (5)	0.0427 (6)	0.0472 (6)	0.0054 (5)	0.0092 (5)	−0.0268 (5)
C11	0.0187 (6)	0.0189 (7)	0.0189 (6)	0.0009 (5)	0.0059 (5)	0.0001 (5)
C19	0.0188 (6)	0.0189 (7)	0.0170 (6)	0.0031 (5)	0.0089 (5)	0.0019 (5)
C7	0.0200 (6)	0.0141 (6)	0.0150 (6)	0.0016 (5)	0.0063 (5)	0.0008 (5)
C14	0.0171 (6)	0.0177 (7)	0.0142 (6)	−0.0010 (5)	0.0045 (5)	0.0013 (5)
C16	0.0259 (7)	0.0312 (8)	0.0191 (6)	−0.0075 (6)	0.0127 (6)	−0.0023 (6)
C6	0.0208 (7)	0.0240 (8)	0.0207 (7)	−0.0022 (6)	0.0084 (6)	−0.0033 (6)
C12	0.0215 (7)	0.0192 (7)	0.0171 (6)	0.0008 (5)	0.0093 (5)	0.0011 (5)
C4	0.0182 (6)	0.0186 (7)	0.0189 (6)	0.0001 (5)	0.0073 (5)	0.0009 (5)
C8	0.0199 (7)	0.0237 (7)	0.0193 (7)	−0.0004 (6)	0.0084 (5)	0.0003 (5)
C3	0.0166 (6)	0.0195 (7)	0.0191 (6)	0.0013 (5)	0.0066 (5)	−0.0008 (5)
C15	0.0238 (7)	0.0262 (8)	0.0172 (6)	−0.0032 (6)	0.0097 (6)	−0.0044 (6)
C17	0.0204 (7)	0.0213 (7)	0.0188 (6)	−0.0037 (6)	0.0048 (5)	0.0024 (5)
C9	0.0270 (7)	0.0280 (8)	0.0177 (6)	0.0000 (6)	0.0113 (6)	0.0009 (6)
C21	0.0495 (11)	0.0482 (11)	0.0253 (8)	−0.0297 (9)	0.0188 (8)	−0.0057 (7)

### Geometric parameters (Å, °)

**Table:**

Cl13—C10	1.7461 (15)	C19—C14	1.3892 (19)
N2—C3	1.3307 (18)	C19—H19	0.9500
N2—N1	1.3605 (15)	C7—C8	1.3924 (19)
O20—C17	1.3714 (18)	C7—C12	1.3998 (19)
O20—C21	1.4312 (19)	C14—C15	1.383 (2)
F3—C6	1.3308 (17)	C16—C17	1.385 (2)
F2—C6	1.3345 (17)	C16—C15	1.393 (2)
N1—C5	1.3680 (17)	C16—H16	0.9500
N1—C14	1.4305 (17)	C6—C3	1.4884 (19)
C10—C11	1.384 (2)	C12—H12	0.9500
C10—C9	1.385 (2)	C4—C3	1.3962 (19)
C5—C4	1.3807 (19)	C4—H4	0.9500
C5—C7	1.4759 (18)	C8—C9	1.394 (2)
C18—C19	1.381 (2)	C8—H8	0.9500
C18—C17	1.393 (2)	C15—H15	0.9500
C18—H18	0.9500	C9—H9	0.9500
F1—C6	1.3313 (18)	C21—H21A	0.9800
C11—C12	1.3861 (19)	C21—H21B	0.9800
C11—H11	0.9500	C21—H21C	0.9800
			
C3—N2—N1	103.79 (11)	F1—C6—F2	106.06 (12)
C17—O20—C21	116.75 (12)	F3—C6—C3	111.98 (12)
N2—N1—C5	112.22 (11)	F1—C6—C3	112.21 (12)
N2—N1—C14	118.35 (11)	F2—C6—C3	112.80 (13)
C5—N1—C14	129.25 (11)	C11—C12—C7	120.73 (13)
C11—C10—C9	121.85 (13)	C11—C12—H12	119.6
C11—C10—Cl13	118.59 (11)	C7—C12—H12	119.6
C9—C10—Cl13	119.55 (11)	C5—C4—C3	104.46 (12)
N1—C5—C4	106.47 (11)	C5—C4—H4	127.8
N1—C5—C7	124.14 (12)	C3—C4—H4	127.8
C4—C5—C7	128.73 (12)	C7—C8—C9	120.73 (13)
C19—C18—C17	120.10 (13)	C7—C8—H8	119.6
C19—C18—H18	120.0	C9—C8—H8	119.6
C17—C18—H18	120.0	N2—C3—C4	113.05 (12)
C10—C11—C12	118.89 (13)	N2—C3—C6	118.86 (12)
C10—C11—H11	120.6	C4—C3—C6	127.88 (13)
C12—C11—H11	120.6	C14—C15—C16	119.99 (13)
C18—C19—C14	119.66 (12)	C14—C15—H15	120.0
C18—C19—H19	120.2	C16—C15—H15	120.0
C14—C19—H19	120.2	O20—C17—C16	124.47 (13)
C8—C7—C12	119.10 (12)	O20—C17—C18	115.32 (13)
C8—C7—C5	120.19 (12)	C16—C17—C18	120.21 (13)
C12—C7—C5	120.48 (12)	C10—C9—C8	118.70 (13)
C15—C14—C19	120.45 (13)	C10—C9—H9	120.6
C15—C14—N1	119.79 (12)	C8—C9—H9	120.6
C19—C14—N1	119.76 (12)	O20—C21—H21A	109.5
C17—C16—C15	119.53 (13)	O20—C21—H21B	109.5
C17—C16—H16	120.2	H21A—C21—H21B	109.5
C15—C16—H16	120.2	O20—C21—H21C	109.5
F3—C6—F1	106.49 (12)	H21A—C21—H21C	109.5
F3—C6—F2	106.86 (12)	H21B—C21—H21C	109.5
			
C3—N2—N1—C5	−0.10 (14)	C12—C7—C8—C9	0.1 (2)
C3—N2—N1—C14	175.45 (11)	C5—C7—C8—C9	−174.46 (13)
N2—N1—C5—C4	0.92 (15)	N1—N2—C3—C4	−0.79 (15)
C14—N1—C5—C4	−174.02 (12)	N1—N2—C3—C6	174.38 (12)
N2—N1—C5—C7	−170.48 (12)	C5—C4—C3—N2	1.34 (16)
C14—N1—C5—C7	14.6 (2)	C5—C4—C3—C6	−173.29 (13)
C9—C10—C11—C12	0.3 (2)	F3—C6—C3—N2	143.23 (13)
Cl13—C10—C11—C12	−178.35 (11)	F1—C6—C3—N2	−97.07 (16)
C17—C18—C19—C14	−2.7 (2)	F2—C6—C3—N2	22.64 (18)
N1—C5—C7—C8	−147.72 (14)	F3—C6—C3—C4	−42.4 (2)
C4—C5—C7—C8	42.9 (2)	F1—C6—C3—C4	77.29 (18)
N1—C5—C7—C12	37.8 (2)	F2—C6—C3—C4	−163.00 (14)
C4—C5—C7—C12	−131.61 (15)	C19—C14—C15—C16	1.4 (2)
C18—C19—C14—C15	1.0 (2)	N1—C14—C15—C16	−178.04 (12)
C18—C19—C14—N1	−179.52 (12)	C17—C16—C15—C14	−2.2 (2)
N2—N1—C14—C15	45.45 (17)	C21—O20—C17—C16	0.4 (2)
C5—N1—C14—C15	−139.87 (14)	C21—O20—C17—C18	179.96 (14)
N2—N1—C14—C19	−134.02 (13)	C15—C16—C17—O20	180.00 (13)
C5—N1—C14—C19	40.7 (2)	C15—C16—C17—C18	0.5 (2)
C10—C11—C12—C7	−0.6 (2)	C19—C18—C17—O20	−177.60 (12)
C8—C7—C12—C11	0.4 (2)	C19—C18—C17—C16	2.0 (2)
C5—C7—C12—C11	174.95 (13)	C11—C10—C9—C8	0.2 (2)
N1—C5—C4—C3	−1.30 (15)	Cl13—C10—C9—C8	178.84 (11)
C7—C5—C4—C3	169.57 (13)	C7—C8—C9—C10	−0.4 (2)
